# A *Clostridioides difficile* cell-free gene expression system for prototyping and gene expression analysis

**DOI:** 10.1128/aem.01566-24

**Published:** 2024-12-31

**Authors:** Ji Zeng, Hao Wang, Yuxi Xu, Jianying Han, Yannan Li, Shu'an Wen, Changbu Wu, Dani Li, Zheng Liu, Xiaokang Zhang, Guo-Bao Tian, Min Dong

**Affiliations:** 1 School of Biomedical and Pharmaceutical Sciences, Guangdong University of Technology 571332 , Guangzhou, Guangdong, China; 2 Department of Microbiology, Zhongshan School of Medicine, Sun Yat-Sen University 626118 , Guangzhou, Guangdong, China; 3 Advanced Medical Technology Center, The First Affiliated Hospital, Zhongshan School of Medicine, Sun Yat-sen University 74644 , Guangzhou, Guangdong, China; 4 Key Laboratory of Tropical Diseases Control, Sun Yat-sen University, Ministry of Educat­ion 26469 , Guangzhou, Guangdong, China; 5 Kobilka Institute of Innovative Drug Discovery, School of Medicine, The Chinese University of Hong Kong 407605 , Shenzhen, Guangdong, China; 6 Shenzhen Institute of Advanced Technology, Chinese Academy of Sciences 85411 , Shenzhen, Guangdong, China; 7 Department of Microbiology, Harvard Medical School 1811 , Boston, Massachusetts, USA; 8 Department of Urology, Boston Children’s Hospital, Harvard Medical School 1811 , Boston, Massachusetts, USA; Centers for Disease Control and Prevention, Atlanta, Georgia, USA

**Keywords:** *Clostridioides difficile*, CFE system, *in vitro *expression, prototyping, *tcdR *and* tcdB *expression

## Abstract

*Clostridioides difficile* is an obligate anaerobic, Gram-positive bacterium that produces toxins. Despite technological progress, conducting gene expression analysis of *C. difficile* under different conditions continues to be labor-intensive. Therefore, there is a demand for simplified tools to investigate the transcriptional and translational regulation of *C. difficile*. The cell-free gene expression (CFE) system has demonstrated utility in various applications, including prototyping, protein production, and *in vitro* screening. In this study, we developed a *C. difficile* CFE system capable of *in vitro* transcription and translation (TX-TL) in the presence of oxygen. Through optimization of cell extract preparation and reaction systems, we increased the protein yield significantly. Furthermore, our observations indicated that this system exhibited higher protein yield using linear DNA templates than circular plasmids for *in vitro* expression. The prototyping capability of the *C. difficile* CFE system was assessed using a series of synthetic *Clostridium* promoters, demonstrating a good correlation between *in vivo* and *in vitro* expression. Additionally, we tested the expression of *tcdB* and *tcdR* from clinically relevant *C. difficile* strains using the CFE system, confirming higher toxin expression of the hypervirulent strain R20291. We believe that the CFE system can not only serve as a platform for *in vitro* protein synthesis and genetic part prototyping but also has the potential to be a simplified model for studying metabolic regulations in *Clostridioides difficile*.

**IMPORTANCE:**

*Clostridioides difficile* has been listed as an urgent threat due to its antibiotic resistance, and it is crucial to conduct gene expression analysis to understand gene functionality. However, this task can be challenging, given the need to maintain the bacterium in an anaerobic environment and the inefficiency of introducing genetic material into *C. difficile* cells. Conversely, the *C. difficile* cell-free gene expression (CFE) system enables *in vitro* transcription and translation in the presence of oxygen within just half an hour. Furthermore, the composition of the CFE system is adaptable, permitting the addition or removal of elements, regulatory proteins for example, during the reaction. As a result, this system could potentially offer an efficient and accessible approach to accelerate the study of gene expression and function in *Clostridioides difficile*.

## INTRODUCTION

*Clostridioides difficile* infection (CDI) has been classified as an urgent antibiotic resistance threat by the Centers for Disease Control and Prevention (1, 2). Annually, it is responsible for an estimated 0.25–0.5 million infection cases in the U.S., leading to 13,000–20,000 in-hospital deaths (1–3). Clinical manifestations of CDI span from mild diarrhea to severe conditions, such as pseudomembranous colitis and toxic megacolon (4). With the process of globalization, CDI has emerged as a significant global health concern, particularly in developed nations (4, 5). Addressing the challenge posed by CDI requires the implementation of therapeutic interventions and the development of strategies for disease control. Gene expression analysis is crucial in characterizing gene expression and function, particularly in the essential process of deleting specific genes and observing their impact on another gene. A few systems have been developed for genetic modification, such as the Clostron system, the Allele-Coupled Exchange systems, and the CRISPR systems (6–8).

Despite numerous technological advancements, gene expression analysis remains a time-consuming and challenging endeavor. Firstly, the process of introducing genetic material, e.g., for genome editing or reporter gene expression analyses, into *C. difficile* is inefficient and typically requires inter-species conjugation (6–8). Secondly, the shuttle plasmid for conjugation requires two sets of replication and antibiotic markers, making it large and laborious to construct. Thirdly, cultivating *C. difficile* requires anaerobic conditions and results in a relatively slow rate of bacterial growth, taking 5–7 hours to reach the mid-log phase and much longer to reach the same optical density (OD) value with a plasmid. Fourthly, genome editing relies on good nicking loci and/or efficient homologous recombination arm, which usually requires pre-screening. Finally, gene expression levels often fall below the detection threshold of measurement devices, leading to a misunderstanding of gene functions. Therefore, there is a pressing need for more efficient and convenient tools for *C. difficile* transcription and translation regulatory study.

The cell-free gene expression system (CFE) reconstructs vital live processes in a test tube, free from the boundary of a cell membrane (9). This system is created by integrating components for transcription and translation, such as amino acids and dNTP, into the cell lysate, which retains the fundamental machinery required for these processes (9). CFE was initially designed for *in vitro* protein synthesis but has since been extended to various applications, such as prototyping, bio-sensing, refining metabolic pathways, and constructing artificial cells (9–12). An intriguing characteristic of cell-free systems is their openness and ease of design, enabling the introduction of different combinations of elements, such as RNA polymerase and regulatory proteins, into the system without the constraints of a cellular membrane. Additionally, the absence of live cell constraints enables cell-free gene expression systems to exhibit non-native properties, expression of toxic proteins for example, without interference from cellular metabolites.

In this study, we have developed a robust, high-yield, and convenient cell-free expression system based on *Clostridioides difficile*. To achieve robust and high-yield protein synthesis, we optimized the process of preparing cell extracts. Subsequently, we optimized the system and increased the protein yield significantly through systematic adjustments to the concentrations of various components. Following this, we assessed this system by measuring the strength of six synthetic promoters with known intensity in *Clostridium acetobutylicum* (13). The *in vitro* and *in vivo* expression trends of these promoters in *C. difficile* were consistent (13). Finally, the system was employed to evaluate the activity variation of the toxin gene *tcdB* and the sigma factor *tcdR* in different clinically relevant *Clostridioides difficile* strains, demonstrating the higher toxin production of the hypervirulent strain R20291 (14, 15). The entire CFE system construction and testing time can be finished in 2–5 days; therefore, our CFE system has the potential to become a valuable tool for *Clostridioides difficile* investigation.

## RESULTS

### Establishment of the *C. difficile* cell-free expression system

**Fig 1:**
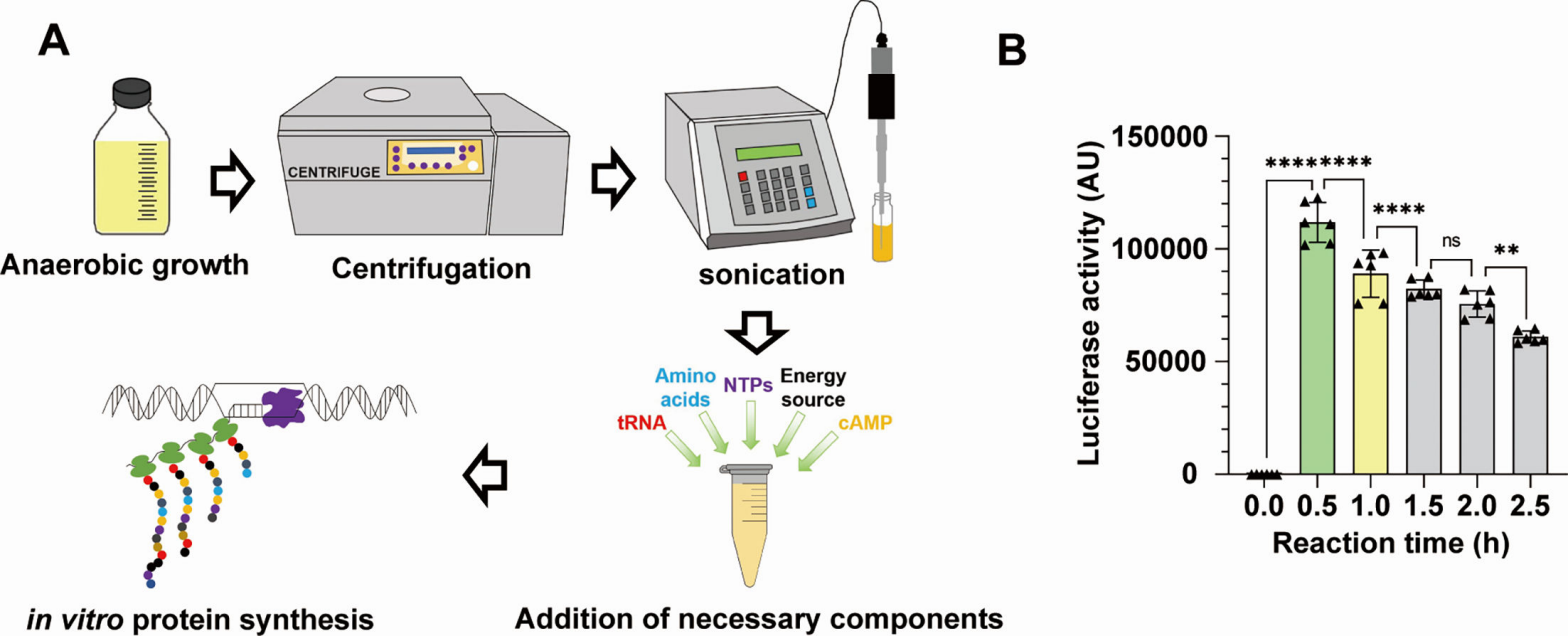
Establishment of the *Clostridioides difficile* cell-free expression system. (**A**) A simplified diagram outlining the preparation of the CFE reaction. *C. difficile* cells were anaerobically inoculated in BHIS medium, then subjected to centrifugation, sonication, and necessary processes to produce the extract. This extract was then supplemented with the energy buffer, amino acids, and other necessary components to establish the complete CFE system. Subsequently, the DNA template was introduced into the CFE system to initiate *in vitro* protein synthesis. (**B**) Quantification of protein yield at different time points. The luciferase activity was measured at 0, 0.5, 1.0, 1.5, 2.0, and 2.5 hours with the highest luciferase activity observed at 0.5 hour. (*P* values were calculated by one-way analysis of variance [ANOVA], ****P ≤ 0.0001, ***P ≤ 0.001, **P ≤ 0.01, *P ≤ 0.05.)

In order to establish a robust and high-yielding *C. difficile* CFE system, we utilized the well-established *Escherichia coli*-based CFE system as a reference, making necessary modifications (16). The basic process is outlined in Fig. 1A, such that *C. difficile* was anaerobically inoculated in brain heart infusion-supplemented (BHIS) medium and subsequently resuspended aerobically in S30A buffer (16). The cells were then lysed using sonication, followed by centrifugation to harvest the extract, which was then supplemented with the previously described energy buffer to complete the CFE system (16).

For testing the transcription and translation (TX-TL) efficiency of the *C. difficile* CFE system, we chose firefly luciferase as the reporter (17). Specifically, the gene of *luciferase* was linked to the T7 promoter, and T7 RNA polymerase was externally introduced into the reaction system. The activity of the CFE system was then measured based on the expression of *luciferase*. To determine the optimal CFE reaction time, the luciferase activity in the CFE system was monitored over a period of 0–2.5 hours at 37°C (Fig. 1B; Table S1). The highest luciferase activity was observed at 0.5 hours, with luminescence declining thereafter, indicating energy depletion and the accumulation of metabolic by-products.

**Fig 2:**
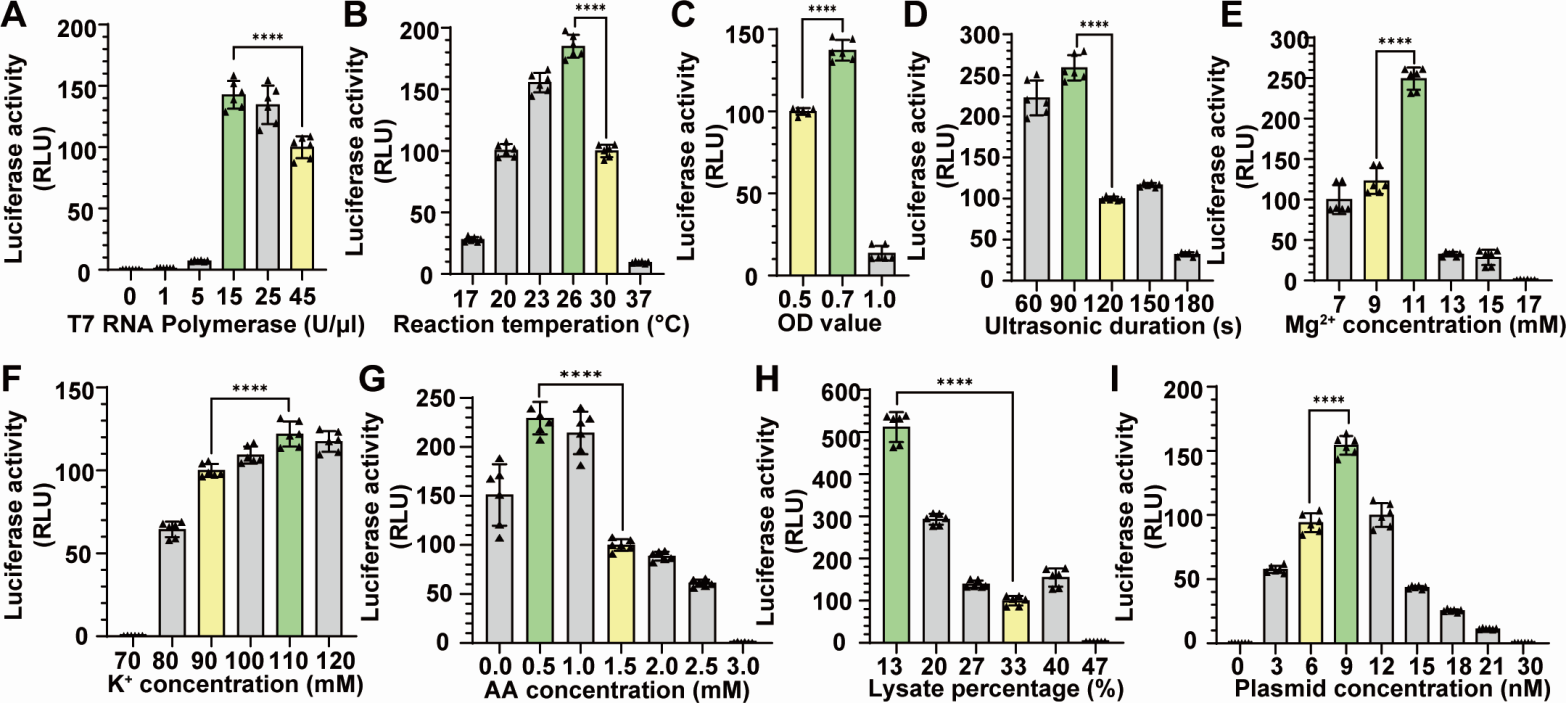
Optimization of Clostridioides difficile extract preparation and CFE reaction conditions. (A) The extract preparation was optimized by evaluating T7 RNA Ppolymerase concentrations of 0, 1, 15, 25, and 45 U/µL, with the highest luciferase activity observed at 15 U/µL. In addition, the extract was optimized by testing different reaction temperatures (B), OD values (C), and ultrasonic durations (D). The CFE reaction condition was optimized by testing a range of Mg2+ concentrations (E), K+ concentrations (F), AA (amono acid) concentrations (G), extract percentages (H), and plasmid concentrations (I). (pP values were calculated by one-way ANOVA, ****P ≤ 0.0001, ***P ≤ 0.001, **P ≤ 0.01, *P ≤ 0.05).

After setting the reaction time at 0.5 hours, our next objective was to optimize the T7 RNA polymerase concentration. Initially, 45 U/µL T7 RNA polymerase was incorporated into the reaction system to guarantee sufficient luciferase production during the establishment of the CFE system (Fig. 1B; Table S1). However, recognizing the energy and resource constraints inherent in the CFE system, optimizing the transcription-to-translation ratio was deemed essential for enhancing protein yield. Consequently, a gradient of T7 RNA polymerase concentrations was evaluated, revealing that 15 U/µL of the enzyme exhibited the highest luciferase activity (Fig. 2A).

Subsequently, we investigated the optimal CFE reaction temperature. To assess this, we established a temperature gradient ranging from 17°C to 37°C (Fig. 2B). The data indicated that the optimized temperature was 26°C, which is lower than the optimized *C. difficile* growth condition of 37°C. This phenomenon has been observed in other TX-TL CFE systems (18, 19). A plausible explanation is that the optimal temperature for proteases and RNases is 37°C; therefore, a lower temperature may result in slower degradation of RNA transcripts and their corresponding proteins, ultimately leading to increased protein yield. Consequently, we have established the *C. difficile* cell-free expression system utilizing 15 U/µL T7 RNA polymerase, a 0.5-hour reaction time, and a 26°C reaction temperature.

### Optimization of the extract preparation

To enhance TX-TL efficiency, we meticulously optimized several critical factors in extract preparation, including the OD of *C. difficile* cells, sonication time, runoff, and dialysis. The CFE system is typically derived from cells during the exponential phase, representing the peak of metabolic activity during growth (18, 19). Therefore, we sought to determine the ideal culturing time for extract preparation. Bacteria were cultured to OD600 values of 0.5, 0.7, and 1.0, and the luciferase activity of CFE produced from each OD value was compared (Fig. 2C). The results revealed that bacteria reaching an OD600 of 0.7 exhibited the highest protein yield, approximately 50% more than those at an OD600 of 0.5.

We posited that sonication time would also impact the activity of the *C. difficile* extract, aiming for an optimized duration that would fully lyse the bacterial cell membrane while preserving the integrity of the enzymes within the extract. To identify the most suitable ultrasonic conditions, a range of sonication times was tested (Fig. 2D). Notably, bacterial extract subjected to 90 seconds of sonication demonstrated the highest luciferase activity. As expected, prolonged ultrasonic treatment compromised CFE activity.

Two subsequent steps were anticipated to enhance protein synthesis following sonication: runoff and dialysis (20). Runoff involves incubating the extract at 37°C with agitation, facilitating protein synthesis as ribosomes “runoff” native mRNAs, which are subsequently degraded by endogenous RNases (16). Conversely, dialysis aims to eliminate small inhibitory molecules during extract composition alteration (20). Our data indicated that runoff did not enhance luciferase expression, as those subjected to the runoff step lost their protein expression ability (Fig. S1A). Similarly, we observed a notable decrease in the expression capacity of the lysate following dialysis (Fig. S1B). Consequently, these two steps were omitted from our protocol.

### Optimization of the *C. difficile* CFE reaction conditions

To enhance the CFE system further, we optimized several key components in the reaction, including salt ion concentration, amino acid concentration, extract percentage, and DNA template concentration. Magnesium ions (Mg^2+^) are crucial for maintaining the functionality of numerous enzymes, such as RNA polymerase, DNA polymerase, and all ATP-utilizing enzymes (21). Therefore, we conducted tests with magnesium glutamate concentrations ranging from 0 mM to 13 mM (Fig. 2E). Our data indicated that the optimized Mg concentration was 11 mM, closely resembling that of the *E. coli* CFE system (16). Subsequent increases in magnesium ion concentration led to a marked reduction in luciferase production. Potassium, the most abundant bacterial cation, is involved in pH homeostasis, protein synthesis, and enzyme activation (22). We optimized the potassium concentration by testing potassium glutamate concentrations between 80 mM and 120 mM (Fig. 2F). Our experiments revealed that maximal *luciferase* expression occurred at 110 mM potassium glutamate.

After optimizing the salt ion concentration, we evaluated luciferase expression in the CFE reaction at varying amino acid concentrations (Fig. 2G). Our data showed that concentrations exceeding 0.5 mM amino acids gradually decreased CFE activity. Unexpectedly, *luciferase* was expressed in the absence of externally added amino acids, indicating a higher intracellular amino acid concentration in *C. difficile* compared to *E. coli* and other established CFE systems (16, 20).

The extract percentage of the entire CFE reaction has been rarely explored in established CFE systems (16, 18–20, 23). We suspected a delicate balance between the extract and externally added energy buffer and components. Consequently, we varied the *C. difficile* extract percentage in volume to examine optimized luciferase expression (Fig. 2H), revealing that 13% extract in the reaction system exhibited maximized activity. Similarly, the plasmid concentration required optimization to prevent excessive energy and resources from being wasted in producing extra mRNAs. Our data showed that the highest CFE expression was achieved at 9 nM among the tested 0–30 nM plasmid concentrations (Fig. 2I).

### Evaluation of the linear DNA fragment as a template for CFE reaction

**Fig 3:**
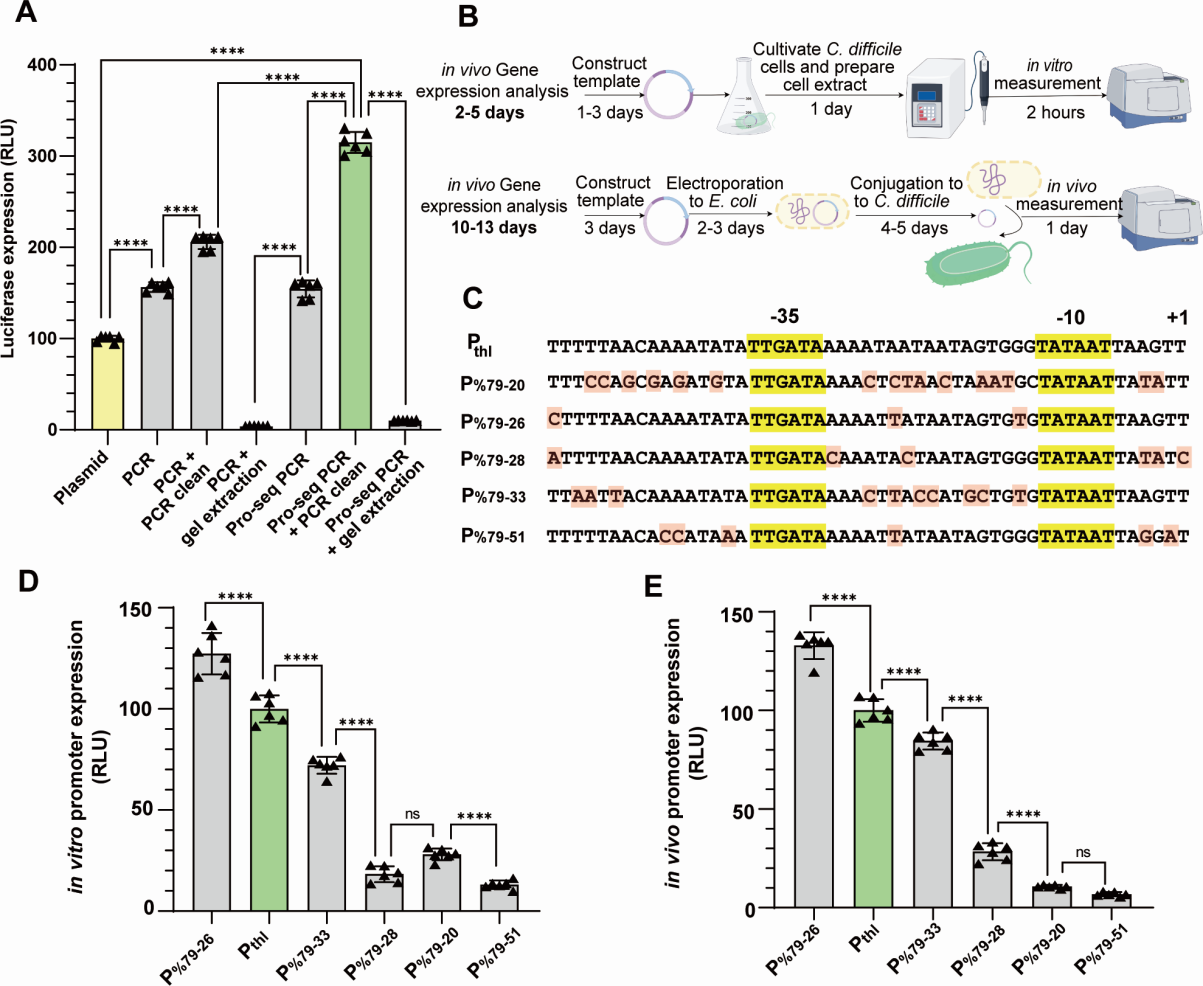
(**A**) *In vitro* expression of different DNA forms as templates for CFE reaction. Equal concentrations of DNA templates were examined, including plasmid, PCR product (PCR), PCR followed by PCR cleanup (PCR + PCR clean), PCR followed by gel extraction (PCR + gel extraction), PCR product with protective sequence (Pro-seq PCR), Pro-seq PCR followed by PCR cleanup (Pro-seq PCR + PCR clean), and Pro-seq PCR followed by gel extraction. The greatest luciferase activity was detected in the case of Pro-seq PCR + PCR clean. (**B**) Comparison of time required to measure gene expression *in vitro* and *in vivo*. The preparation of templates for *in vitro* expression analysis may only take 1 day, as the template could be linear PCR product, bypassing the laborious plasmid construction and sequencing. Moreover, the cultivation and measurement time for *in vitro* expression were also reduced. The figure was drawn by Figuredraw (https://www.figdraw.com/). (**C**) The sequences of *Clostridium* promoters for *in vitro* prototyping (**D**) and i*n vivo* expression analysis (**E**). The −35 and −10 regions were indicated above the alignment. The single base variation was red-colored. The patterns of the promoter intensity were comparable for both *in vivo* and *in vitro* analyses. (*P* values were calculated by one-way ANOVA, ****P ≤ 0.0001, ***P ≤ 0.001, **P ≤ 0.01, *P ≤ 0.05.)

Following the optimization of the CFE system, our subsequent object was to demonstrate its application in genetic part prototyping. An obstacle in this application was the laborious molecular cloning and plasmid template construction. As a result, we sought to evaluate the gene expression of linear DNA templates, aiming to expedite the prototyping process. To this end, we compared the expression of PCR products to a plasmid template in the CFE reaction system (Fig. 3A). Surprisingly, the PCR product outperformed the plasmid as a CFE reaction template, yielding a 1.5 times higher *luciferase* expression compared to the plasmid template, potentially due to a low intracellular concentration of nucleases. Furthermore, PCR cleanup, directly purifying PCR reaction without running an agarose gel, notably enhanced the efficiency of the PCR product, likely due to the removal of unfavorable pH, excessive enzymes, and dNTPs. However, gel extraction significantly reduced *luciferase* expression from the PCR product, indicating template damage during the purification process, or the possibility that the gel extraction process introduces substances that hinder the *luciferase* reactions.

The challenge with PCR templates lies in their instability in the CFE reaction. Thus, a previous study identified a 20 bp sequence with 65% guanine-cytosine (GC) content, which could enhance the protein synthesis rate of the linear DNA template (24). Consequently, we incorporated this 20 bp sequence at both ends of our PCR product template to assess its protective effects (Fig. 3A). It was observed that the protein yield from the PCR product plus protective sequences (PPPPS) was comparable to that without protective sequences. However, the protein yield of PPPPS after PCR cleanup markedly increased, suggesting that the sequences not only protected the linear template from DNase but also from physical and chemical damage during PCR cleanup.

### Prototyping of *C. difficile* genetic parts

Prototyping involves using a cell-free gene expression system to evaluate genetic components such as promoters and RBS strength before integrating them into living systems (25). This approach significantly reduces the time needed for investigation. For instance, in the case of *C. difficile*, the process from template construction to prototyping only required 2–5 days, depending on the template type, whereas *in vivo* measurements took 10–13 days (Fig. 3B). Despite its convenience, it is important to note that the strength of a promoter can vary significantly across different bacterial species. As a result, it is essential to establish a species-specific cell-free expression system for each bacterium (13, 26, 27).

Due to the absence of appropriate *Clostridioides difficile* promoters for prototyping, we selected a series of artificial promoters, which displayed a range of activity from high to low in *Clostridium acetobutylicum* (13). We ligated the promoters of *Clostridium* to the *luciferase* gene with the protective sequence added at both ends, and the derived linear DNA fragments were used as the template for promoter *in vitro* expression (Fig. 3C). The strong constitutive thiolase gene promoter was set as the reference (13), and we observed variations in promoter strength due to changes in DNA sequence surrounding the −10 and −35 regions (Fig. 3D). To confirm that the promoter activity was induced by native RNA polymerase, rifampicin was introduced into the CFE reaction to inhibit the native RNA polymerase (Fig. S2). These promoters were also integrated into the pDSW1728 plasmid, replacing the original P_tet_ promoter (28). The relative expression strength of these promoters was measured by *in vivo* mCherry fluorescence, which was found to be comparable to the *in vitro* expression (Fig. 3E). As a result, the *C. difficile* CFE system shows promise as a tool for prototyping synthetic promoters.

### Transcriptional study by *C. difficile* CFE system

As the *C. difficile* CFE system was approved to be useful as a prototyping tool, our subsequent object was to explore additional applications of this system. Notably, the ribotype 027 (RT027) strains are often described as “hypervirulent” because they have historically been associated with more severe *C. difficile* infections and epidemics than other ribotypes, although the severity of this strain may vary (14, 29). Research has demonstrated that RT027 produces significantly greater quantities of toxins, up to 23 times more TcdB, than other strains during the stationary phases (15). However, the reason for the hypervirulence of RT027 is possibly a consequence of a multifactorial process affected by toxin level, sporulation, application of antibiotics, and antimicrobial resistance (30, 31).

**Fig 4:**
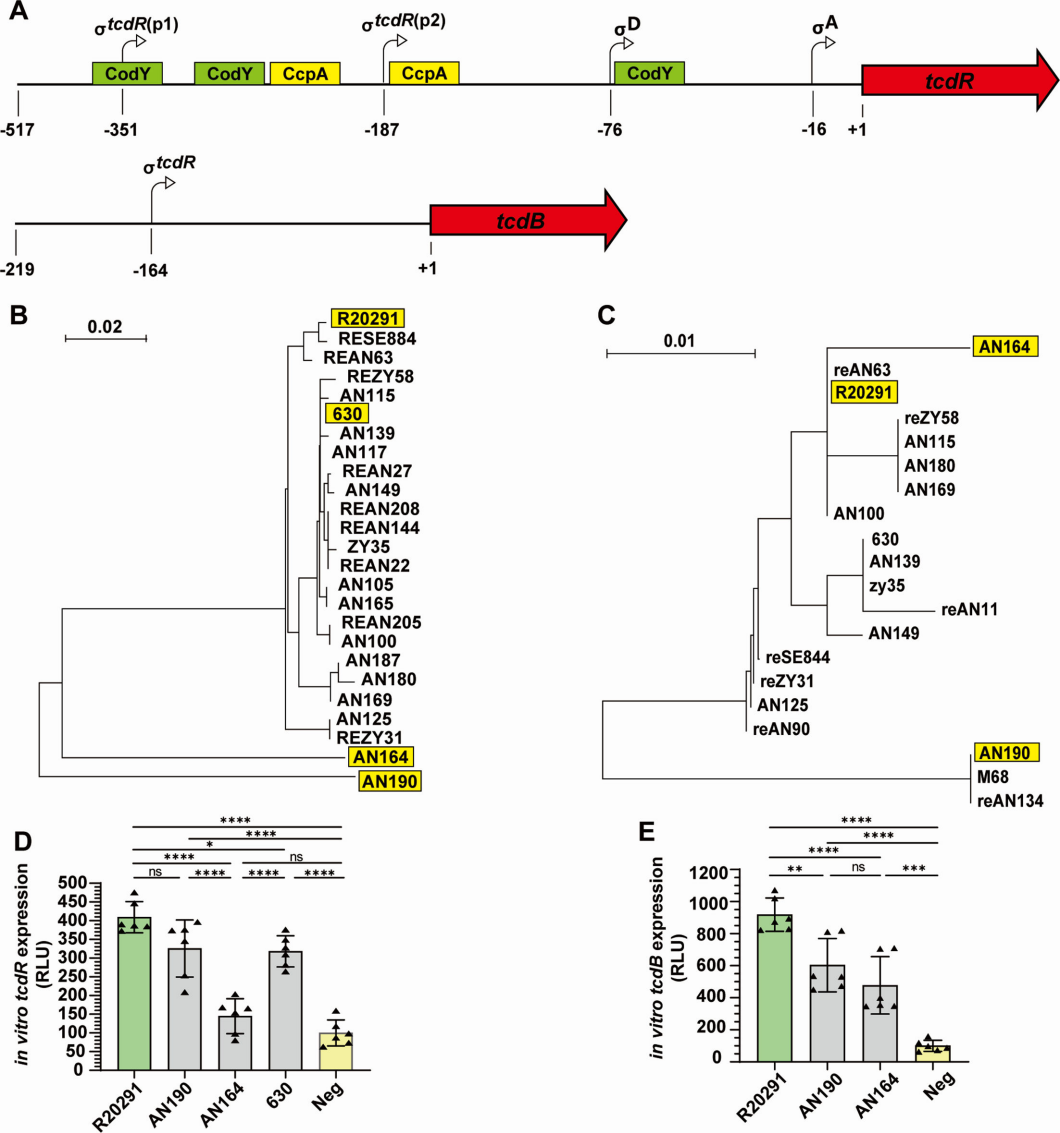
(**A**) The promoter and 5′UTR regions of *tcdR* and *tcdB* with transcriptional start points indicated by arrows and transcriptional factor binding sites marked by boxes. The phylogenetic tree of the promoter and 5′UTR regions of *tcdR* (**B**) and *tcdB* (**C**) were generated by MEGA software (https://www.megasoftware.net/). Based on phylogenetic trees, *tcdR* could be classified into four groups, while *tcdB* could be classified into three groups. The *in vitro* expression levels of the promoter and 5′UTR regions of *tcdR* (**D**) and *tcdB* (**E**) from the representative strains were quantified. The hypervirulent strain R20291 produced significantly more TcdR than AN164 and 630 strains, and significantly more TcdB than AN190 and AN164 strains. The negative control consisted of the CFE reaction without a template. (*P* values were calculated by one-way ANOVA, ****P ≤ 0.0001, ***P ≤ 0.001, **P ≤ 0.01, *P ≤ 0.05.)

The expression of toxins is primarily activated by TcdR, a self-regulated and toxin-specific sigma factor, during the stationary phase (Fig. 4A) (32–36). Nevertheless, the housekeeping expression of toxins during the exponential phase may confer a competitive advantage to *C. difficile*, underscoring the importance of studying the constitutive expression of *tcdB* and *tcdR* (1). As far as we know, there has been no comparative analysis of *tcdR and tcdB* expression across different strains during the exponential phase, likely because of the low toxin expression at this stage. Our *C. difficile* CFE system offers an exceptional platform for comparing the expression of PaLoc genes during the exponential phase, given that the adjustable template concentration could effectively amplify weak signals that might not be detectable through *in vivo* methods.

We collected the promoter and the 5′UTR regions of *tcdR* and *tcdB* from R20291 and 19 clinically relevant strains (Fig. 4A; Fig. S3 and S4). Based on the alignments and phylogenetic trees, the promoter and 5′UTR regions of *tcdR* could be categorized into R20291 group, 630 group, AN164 group, and AN190 group, while *tcdB* could be categorized into AN164 group, AN190 group, and R20291 group (Fig. 4B and C; Fig. S3 and S4). The representative sequence of each group was tested in the CFE system (Fig. 4D and E). Our experiments revealed that the hypervirulent strain R20291 exhibits significantly higher *tcdR* and *tcdB* expression than other strains (with the exception of *tcdR* expression to AN190) during the exponential stage, potentially contributing to the hypervirulence of RT027 strains. In the *in vivo* assay, the expression of the same promoter and 5UTR sequences was indistinguishable from the negative control, likely due to low gene expression during the exponential growth phase (Fig. S5).

## DISCUSSION

*Clostridioides difficile*, a strictly anaerobic pathogen known for its toxin production, necessitates the study of its metabolic networks' transcriptional and translational regulations to identify drug targets and develop effective therapies. Such investigations require measurements of gene expression under various conditions. However, these operations are cumbersome, involving molecular cloning, transformation of shuttle plasmids into *E. coli*, conjugation of shuttle plasmids to *C. difficile*, anaerobic culture, and gene expression measurement. To bypass these challenges, we have developed a *C. difficile*-based TX-TL coupled cell-free gene expression system. This system preserves transcription and translation capabilities without living cells and offers several advantages over traditional *in vivo* methods for studying transcriptional and translational regulation.

The *C. difficile* CFE system has few obvious advantages compared to living cells. Firstly, the TX-TL coupled reactions can be conducted in the presence of oxygen, obviating the need for an inconvenient anaerobic chamber. Secondly, the *in vitro r*eactions are typically completed within half an hour, saving considerable time compared to the hours or days required for bacterial growth. Thirdly, the need for plasmid construction can be eliminated, as the system can utilize a linear PCR product as the template. Fourthly, the template concentration could be adjusted, which could amplify the weak signals that were undetectable in the *in vivo* measuring systems. Last but not least, our system has the potential to partially replace the arduous genetic modification process, such that one or a few elements, e.g., TcdR and other sigma factors, could be added to the system to mimic various genetic backgrounds.

We identified disparities between the *C. difficile* CFE system and other established CFE systems. The reaction necessitates no exogenous amino acid addition. Unlike other systems, which typically required 1.5–2 mM amino acids to achieve optimal protein yield, the high intracellular amino acid concentration was not observed in our system (18, 19). This divergence may be attributed to Stickland fermentations in *Clostridium*, where amino acids serve as carbon, nitrogen, and energy sources (37). Furthermore, it has been documented that a protein-rich diet enhances the amino acid concentration, leading to increased mortality in *C. difficile*-infected mice (38).

Additionally, the *C. difficile* CFE system exhibits strong compatibility with linear DNA templates. This phenomenon may be attributed to *C. difficile*’s low GC content, resulting in relatively low intracellular nucleases (39). Studies have linked high GC content in prokaryotes to increased double-strand DNA breakage and the non-homologous end joining DNA double-strand break repair pathway (40). Moreover, the presence of high-GC sequences at the ends of linear templates has been shown to significantly enhance gene expression in cell-free gene expression systems, likely due to protective effects against native nucleases (24). Interestingly, the addition of a protective sequence at the end of PCR products did not elevate the expression of PCR products in our *C. difficile* CFE system, indicating relatively low native nucleases. However, it did enhance the expression of PCR products subjected to a PCR clean, suggesting an, as of yet, unidentified protective mechanism. We conducted experiments to examine the coupled transcription and translation of t*cdB* and *tcdR* in both the CFE system and live *C. difficile* cells. Our data revealed that the hypervirulent strain R20219 exhibited notably higher expression of *tcdR and tcdB* during the exponential phase of bacterial growth. Through *tcdB* promoter alignment and phylogenetic analysis, we were able to roughly categorize the promoters into three groups: AN164 group, AN190 group, and R20291 group. The most notable disparity among these groups is the presence of an AT-rich element at positions −34 to −22 within the 5′UTR region. The functional significance of this element warrants further investigation.

In contrast, the *tcdR* promoter and 5′UTR regions are more intricate, since most of the regulation occurs within this region (32–34). Based on our alignment and phylogenetic analysis, *tcdR* groups can be roughly classified into four groups: R20291 group, 630 group, AN164 group, and AN190 group. Similar to *tcdB*, the *tcdR* promoter and 5′UTR of R20291 exhibited higher expression.

The CFE system significantly simplifies the transcription and translation study of *C. difficile*, yet it could be further improved in future studies to make it a better investigation tool. Primarily, its energy and resource capacity are restricted, allowing for time point studies but not continuous ones. Recent advancements in microfluidics-based CFE systems may address this challenge (41). Additionally, the absence of a cell membrane boundary in the CFE system makes it difficult to conduct research on signal transduction and membrane proteins. Recent advancements in artificial cells composed of a lipid monolayer and a cell-free gene expression system may provide insights into addressing this issue (42). Furthermore, the *C. difficile* CFE system could be further enhanced via addition of more reaction components. Studies have shown the system’s potential in constructing more complex genetic or synthetic regulatory networks mimicking different genetic backgrounds (43). In our experiments, we tested the transcription of promoters using externally added T7 RNA polymerase and native RNA polymerase, demonstrating the potential for increased complexity. We anticipate that our *C. difficile* CFE system will not only expand the protein synthesis toolkit for synthetic biology and accelerate the screening and prototype design of gene regulatory elements but also serve as a platform for studying regulatory networks of *C. difficile*, expediting the discovery of new drug targets and therapeutic strategies.

## MATERIALS AND METHODS

### Stains and media

Bacterial strains used in this study are listed in Table S2. *Escherichia coli* DH5ɑ (Takara Bio) was used for molecular cloning to generate plasmids for *in vivo* and *in vitro* expression. *E. coli* CA434 was used as the donor strain for conjugation (44). *Escherichia coli* was cultured in lysogeny broth (LB) medium at 37°C with 30 µg/mL chloramphenicol or 50 µg/mL kanamycin as needed. *Clostridioides difficile* 630 was utilized for the preparation of *C. difficile* CFE system and for *in vivo* expression. *C. difficile* was cultured in an anaerobic chamber in BHIS medium (supplemented with 5 g/L yeast extract and 1 g/L L-cycloserine) at 37°C. For conjugation, the BHIS medium was supplemented with 15 µg/mL thiamphenicol, 250 µg/mL cefovecin, and 8 µg/mL fusidic acid.

### Construction of plasmid and linear CFE template

All the plasmids used in this study are listed in Table S2, and all the primers are listed in Table S3. PCR was performed using Phusion High-Fidelity DNA Polymerase (NEB Catalog# M0530L). Plasmids were constructed using NEBuilder HiFi DNA Assembly Master Mix (NEB Catalog# E2621L). The codon-optimized *luciferaseOpt* and *Clostridium* promoters were custom-synthesized by Genwiz. The promoter and 5′UTR regions of *tcdB* and *tcdR* were PCR-amplified from the *Clostridioides difficile* genome. For the generation of linear templates for *in vitro* expression, the *luciferaseOpt* gene was integrated into pUC19 via Gibson assembly, replacing the original *lacZα* gene. Similarly, T7 promoter, the synthesized *Clostridium* promoters, or the promoter and 5′UTR regions were ligated to pUC19 to replace the original P_lacI_ promoter. The resulting plasmid served as the PCR template, and the promoter and *luciferaseOpt* were PCR-amplified to produce the linear CFE template. For the construction of plasmids for *in vivo* expression, T7 promoter, the synthesized *Clostridium* promoters, or the promoter and 5′UTR regions were integrated into pDSW1728 to substitute the original P_tet_ promoter to generate the template for *in vivo* expression.

### CFE extract preparation

Each gram of cell pellets was re-suspended in 0.8 mL of S30A buffer (50 mM Tris acetic acid, pH 7.7, 14 mM Mg glutamate, 10 mM K glutamate, 2 mM DTT), and then aliquoted to 0.33 mL. The re-suspended cells were lysed using a JY92-IIN Sonicator (Jingxin, Shanghai, China) equipped with a 2 mm diameter probe, employing 12 cycles of 5 seconds ON/5 seconds OFF at a 20 kHz frequency and 27% amplitude. To prevent damage from the heat generated during sonication, the samples were kept in an ice-water bath. The lysates were centrifuged at 12,000 × *g*, 4°C, for 10 minutes. The resulting supernatant was flash-frozen with liquid nitrogen and stored in a −80°C refrigerator until use. For run-off and dialysis, the cleared lysates were incubated at 37°C, 250 rpm for 35 or 70 minutes. The sample was then centrifuged, and the supernatant was loaded into a dialysis cassette (Slide-A-Lyzer 10 k MWCO, Thermo Scientific) and dialyzed in S30B buffer (5 mM Tris acetic acid, pH 8.2, 14 mM Mg glutamate, 60 mM K glutamate, 1 mM DTT) (16) for 2.5 hours at 4°C. The supernatant was cleared by centrifugation at 12,000 × *g*, 4°C for 10 minutes. Finally, the supernatant was collected, flash-frozen in liquid nitrogen, and stored at −80°C until use.

### CFE reaction

The initial CFE reaction contained the following components unless otherwise specified: 50 mM HEPES at pH 8.0, 1.5 mM ATP (Macklin), 1.5 mM GTP (Macklin), 0.9 mM CTP (Macklin), 0.9 mM UTP (Macklin), 0.2 mg/mL *E. coli* tRNA (Roche), 0.26 mM CoA (Aladdin), 0.33 mM NAD (Sigma), 0.75 mM cAMP (Macklin), 0.068 mM folinic acid (Aladdin), 1 mM spermidine (Macklin), 30 mM 3-PGA (APExBIO), 9 mM Mg glutamate (Sigma), 90 mM K glutamate (Macklin), 1.5 mM of each of the 20 amino acids (Macklin), 2% PEG-8000 (Sigma), 6 nM template, and RNAse-free water. The CFE reaction was carried out in a PCR tube (Eppendorf, Catalog# 951010006) with a volume of 20–40 μL and incubated at 30°C for 30 minutes in a PCR machine (Eppendorf S1000). The CFE reaction was transferred to a well of a 384-well plate, Luciferase Reporter Gene Assay Kit (Yeasen, Catalog# 11401ES76) was utilized to measure the luciferase activity, and generated fluorescence was recorded by a plate reader (Shanpu, SuPerMax 3100).

### Bacterial transformation

One milliliter of electro-competent *Escherichia coli* CA434 cells were thawed on ice, mixed with the 100 ng/µL plasmid, and incubated on ice for 15 minutes. The mixture was then transferred into a pre-chilled 0.2 cm electroporation cuvette, ice-bathed for 10 minutes, and subjected to electroporation. The gene pulser was set to the following parameters: 200 Ω, 25 µF, 1.8 kV.

### Bacterial conjugation

The donor cells *E. coli* CA434 and recipient cells *C. difficile* 630 were cultured until the OD600 reached 0.8–1.0. One milliliter of *E. coli* CA434 was centrifuged at 2,841 × *g* for 3 minutes and washed once with sterile LB medium. The cell pellet was then transferred into an anaerobic chamber and mixed with 150 µL of *C. difficile* 630 cells. The mixture was spotted onto pre-reduced BHIS agar plates with 20 µL per spot. Following co-incubation anaerobically at 37°C for 10 hours, cells on the plate were scraped and re-suspended in 2 mL BHIS medium, and 100 µL of the sample was spread onto BHIS agar plates supplemented with 15 µg/mL thiamphenicol, 250 µg/mL cefovecin, and 8 µg/mL fusidic acid, and then incubated at 37°C until *C. difficile* colonies formed on the plate.

### Fluorescent reporter gene expression

*Clostridioides difficile* 630 cells transformed with the reporter plasmid were cultured until the OD600 reached about 0.8. Subsequently, samples were centrifuged at 10,000 × *g* for 2 minutes and washed three times with Phosphate-Buffered Saline (PBS). Following this, the cells were 10-fold diluted, and 200 µL of the sample was transferred to a flat-bottom 96-well titration plate. The samples were then incubated in the dark for 30 minutes. The fluorescence was recorded with excitation at 554 nm, emission at 610 nm, and a gain set to 100.

## SUPPLEMENTAL MATERIAL

Supplemental material aem.01566-24-s0001.docx Figures S1 to S5; Tables S1 to S3.

ASM does not own the copyrights to Supplemental Material that may be linked to, or accessed through, an article. The authors have granted ASM a non-exclusive, world-wide license to publish the Supplemental Material files. Please contact the corresponding author directly for reuse.
