# Supplementary material for: A Clostridioides difficile cell-free gene expression system for prototyping and gene expression analysis
Source: Appl Environ Microbiol. 2024 Dec 31;91(1):e01566-24. doi: 10.1128/aem.01566-24 (PMC11784378; doi:10.1128/aem.01566-24)
Supplement: Supplemental material — Figures S1 to S5; Tables S1 to S3. [file aem.01566-24-s0001.docx]

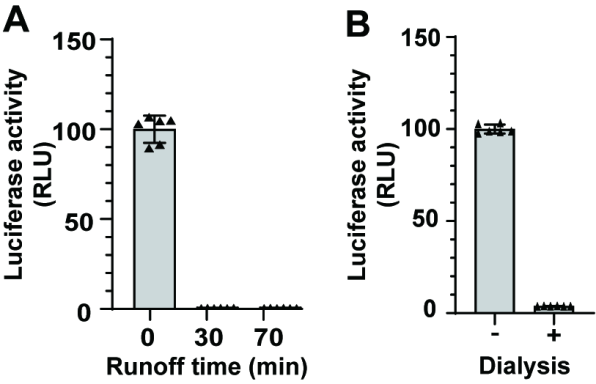


Figure S1 Optimization of the cell free expression system by testing runoff (A) and dialysis (B). The preparation of *C. difficile* lysate without runoff and dialysis exhibited significantly higher luciferase activity compared to the samples with runoff and dialysis, respectively.


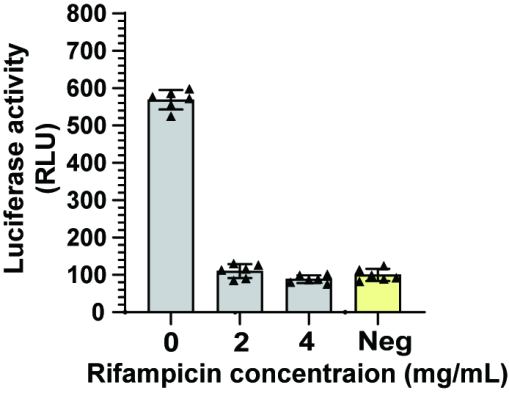


Figure S2 Inhibition of the native RNA polymerase in the CFE reaction by rifampicin. The P_79%-26_ promoter activity in the CFE reaction system was tested in the presence of 0, 2, and 4 mg/mL rifampicin. Negative control consisted the CFE reaction without a template.


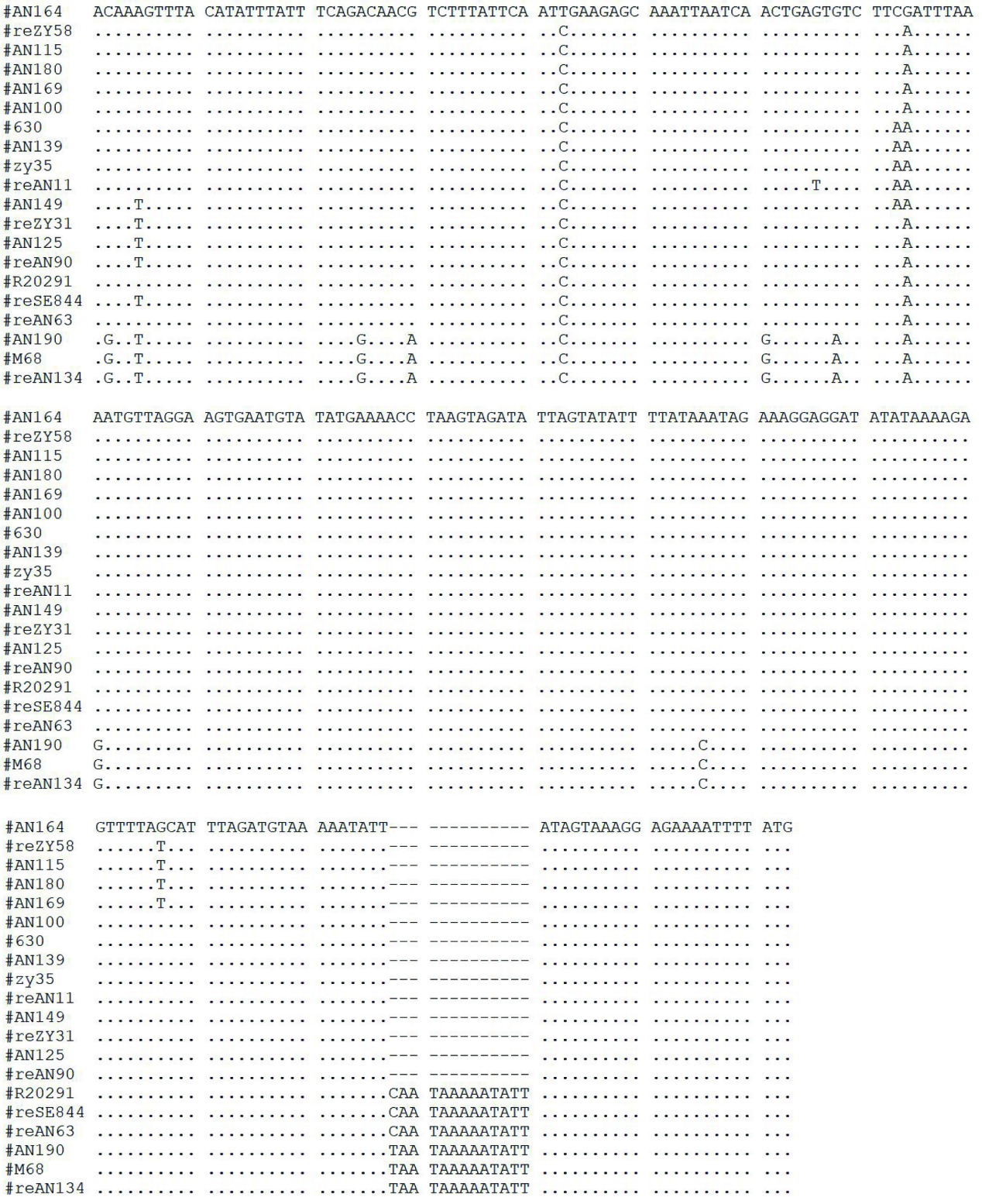


Figure S3 Alignment of the promoter and 5’UTR regions of *tcdB.*


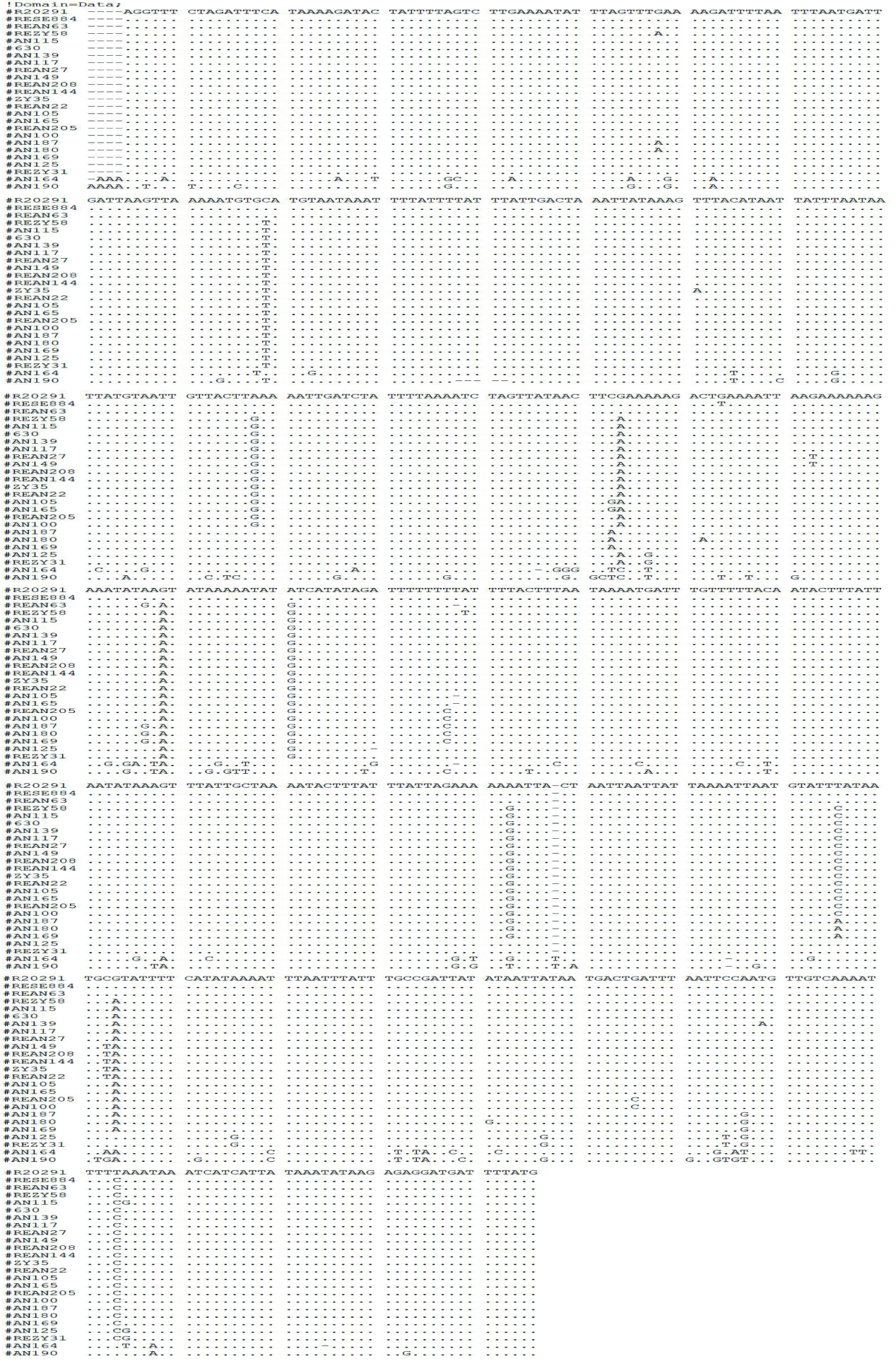


Figure S4 Alignment of the promoter and 5’UTR regions of *tcdR.*


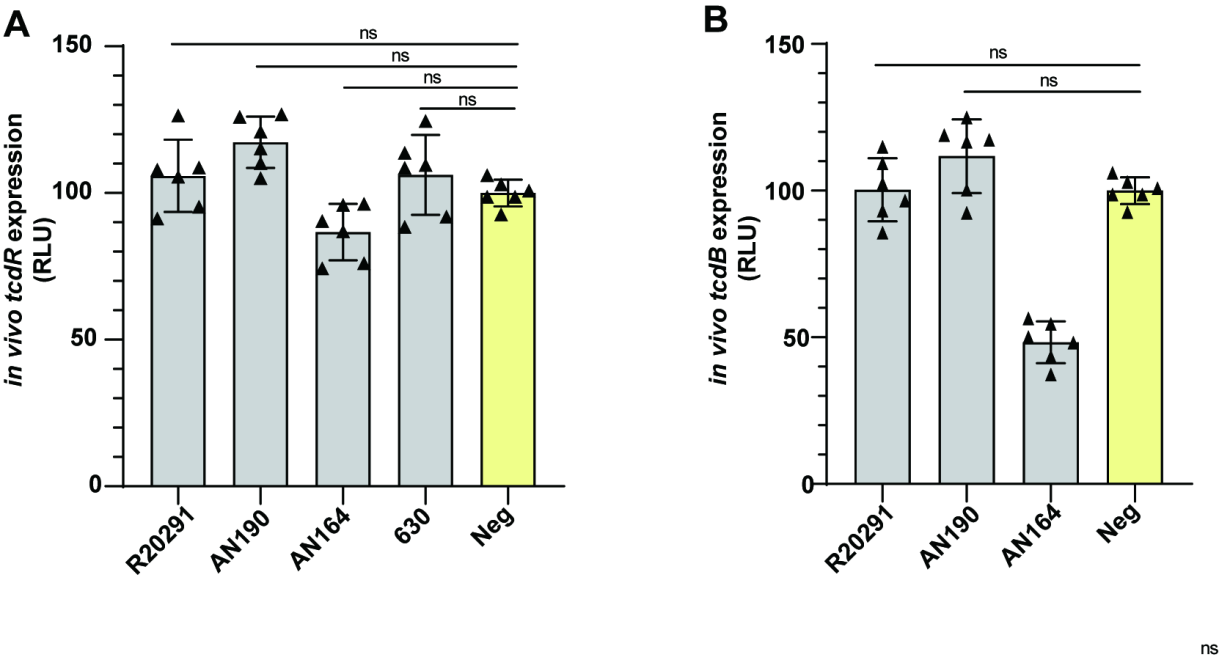


Figure S5 The *in vivo* expression levels of the promoter and 5’UTR regions of *tcdR* (A) and *tcdB* (B) from representative strains. The negative control consisted of the *C. difficile* strain without a reporter plasmid. The expression levels of *tcdR* and *tcdB* were indistinguishable from the negative control.

Supplementary table 1 Optimization summary table

| Corresponding figure | Figure  1B | Figure  2A | Figure  2B | Figure  2C | Figure  2D | Figure  2E | Figure  2F | Figure  2G | Figure  2H | Figure  2I | Final condition |
| --- | --- | --- | --- | --- | --- | --- | --- | --- | --- | --- | --- |
| Optimization steps | Incubation time | T7 RNA polymerase | Temperature | OD_600_ | Ultrasonic time | Mg^2+^  conc. | K^+^  conc. | Amino acid conc. | Lysate | Plasmid conc. |  |
| Before optimization | 1 h | 45 U/μL | 30 ℃ | 0.5 | 120 s | 9 mM | 90 mM | 1.5 mM | 33% | 6 |  |
| After optimization | 0.5 h | 15 U/μL | 26 ℃ | 0.7 | 90 s | 11 mM | 110 mM | 0.5 mM | 13% | 9 |  |
| Incubation time (h) |  | 0.5 | 0.5 | 0.5 | 0.5 | 0.5 | 0.5 | 0.5 | 0.5 | 0.5 | 0.5 |
| T7 RNAP (U/μL) | 45 |  | 15 | 15 | 15 | 15 | 15 | 15 | 15 | 15 | 15 |
| Temperature (℃) | 30 | 30 |  | 26 | 26 | 26 | 26 | 26 | 26 | 26 | 26 |
| OD_600_ | 0.5 | 0.5 | 0.5 |  | 0.7 | 0.7 | 0.7 | 0.7 | 0.7 | 0.7 | 0.7 |
| Ultrasonic time (s) | 120 | 120 | 120 | 120 |  | 90 | 90 | 90 | 90 | 90 | 90 |
| Mg-glutamate (mM) | 9 | 9 | 9 | 9 | 9 |  | 11 | 11 | 11 | 11 | 11 |
| K-glutamate (mM) | 90 | 90 | 90 | 90 | 90 | 90 |  | 110 | 110 | 110 | 110 |
| Amino Acids (mM) | 1.5 | 1.5 | 1.5 | 1.5 | 1.5 | 1.5 | 1.5 |  | 0.5 | 0.5 | 0.5 |
| Lysate (%) | 33 | 33 | 33 | 33 | 33 | 33 | 33 | 33 |  | 13 | 13 |
| Plasmid conc. (nM) | 6 | 6 | 6 | 6 | 6 | 6 | 6 | 6 | 6 |  | 9 |

**Supplemental table 2 Strains and plasmids used in this study**

| **Strain name** | **Genotype and/or description** | **Reference/Source** |
| --- | --- | --- |
| *E. coli* DH5α | F^-^ φ80*lacZ*ΔM15 Δ*(lacZYA*^-^*argF*)U169 *recA*1 *endA*1 *hsdR*17(rK^-^, mK^+^) *phoA* *supE*44 λ^-^*thi*-1 *gyrA*96 *relA*1 | Takara Bio |
| *E. coli* CA434  (HB101 carrying R702) | F^-^ *mcrB* *mrr* *hsdS*20(rB^-^mB^-^) *recA*13 *leuB*6 *ara*^-^13 *proA*2 *lavYI* *galK*2 *xyl*-6 *mtl*-1 *rpsL*20 carrying conjugative plasmid R702 | ^1^ |
| *Clostridioides difficile* 630 | Clinical isolate | From Dr. Liang Tao’s lab1 |
| *Clostridioides difficile* R20291 | Clinical isolate | From Leibniz Institute DSMZ |
| *Clostridioides difficile* AN164 | Clinical isolate | ^2^ |
| *Clostridioides difficile* AN190 | Clinical isolate | ^2^ |
| pDSW1728 | *colE1* *repA* *orfB* *tetR* P_tet_::*mCherryOpt traJ catP* | Addgene |
| pUC19 | *colE1* P_lacI_::*lacZα ampR* | Takara Bio |
| pJZ10 | *colE1* *repA* *orfB* *tetR* P_79%-18_::*mCherryOpt traJ catP* | This study |
| pJZ11 | *colE1* *repA* *orfB* *tetR* P_79%-20_::*mCherryOpt traJ catP* | This study |
| pJZ12 | *colE1* *repA* *orfB* *tetR* P_79%-26_::*mCherryOpt traJ catP* | This study |
| pJZ13 | *colE1* *repA* *orfB* *tetR* P_79%-28_::*mCherryOpt traJ catP* | This study |
| pJZ14 | *colE1* *repA* *orfB* *tetR* P_79%-33_::*mCherryOpt traJ catP* | This study |
| pJZ15 | *colE1* *repA* *orfB* *tetR* P_79%-51_::*mCherryOpt traJ catP* | This study |
| pJZ16 | *colE1* *repA* *orfB* *tetR* P_thl_::*mCherryOpt traJ catP* | This study |
| pJZ17 | *colE1* *repA* *orfB* *tetR* P_tcdB-AN164_::*mCherryOpt traJ catP* | This study |
| pJZ18 | *colE1* *repA* *orfB* *tetR* P_tcdB-AN190_::*mCherryOpt traJ catP* | This study |
| pJZ19 | *colE1* *repA* *orfB* *tetR* P_tcdB-R20291_::*mCherryOpt traJ catP* | This study |
| pJZ20 | *colE1* *repA* *orfB* *tetR* P_tcdR-AN164_::*mCherryOpt traJ catP* | This study |
| pJZ21 | *colE1* *repA* *orfB* *tetR* P_tcdR-AN190_::*mCherryOpt traJ catP* | This study |
| pJZ21 | *colE1* *repA* *orfB* *tetR* P_tcdR-630_::*mCherryOpt traJ catP* | This study |
| pJZ22 | *colE1* *repA* *orfB* *tetR* P_tcdR-R20291_::*mCherryOpt traJ catP* | This study |
| pJZ28 | *colE1* P_T7_::*luciferase ampR* | This study |
| pJZ29 | *colE1* P_79%-18_::*luciferase ampR* | This study |
| pJZ30 | *colE1* P_79%-20_::*luciferase ampR* | This study |
| pJZ31 | *colE1* P_79%-26_::*luciferase ampR* | This study |
| pJZ32 | *colE1* P_79%-28_::*luciferase ampR* | This study |
| pJZ33 | *colE1* P_79%-33_::*luciferase ampR* | This study |
| pJZ34 | *colE1* P_79%-51_::*luciferase ampR* | This study |
| pJZ35 | *colE1* P_thl_::*luciferase ampR* | This study |
| pJZ36 | *colE1* P_tcdB-AN164_::*luciferase ampR* | This study |
| pJZ37 | *colE1* P_tcdB-AN190_::*luciferase ampR* | This study |
| pJZ38 | *colE1* P_tcdB-R20291_::*luciferase ampR* | This study |
| pJZ39 | *colE1* P_tcdR-AN164_::*luciferase ampR* | This study |
| pJZ40 | *colE1* P_tcdR-AN190_::*luciferase ampR* | This study |
| pJZ41 | *colE1* P_tcdR-630_::*luciferase ampR* | This study |
| pJZ42 | *colE1* P_tcdR-R20291_::*luciferase ampR* | This study |

1. Purdy D, O'Keeffe TA, Elmore M, Herbert M, McLeod A, Bokori-Brown M, Ostrowski A, Minton NP. 2002. Conjugative transfer of *clostridial* shuttle vectors from *Escherichia coli* to *Clostridium difficile* through circumvention of the restriction barrier. Mol Microbiol 46:439-52.
2. Wen X, Shen C, Xia J, Zhong LL, Wu Z, Ahmed M, Long N, Ma F, Zhang G, Wu W, Luo J, Xia Y, Dai M, Zhang L, Liao K, Feng S, Chen C, Chen Y, Luo W, Tian GB. 2022. Whole-Genome Sequencing Reveals the High Nosocomial Transmission and Antimicrobial Resistance of *Clostridioides difficile* in a Single Center in China, a Four-Year Retrospective Study. Microbiol Spectr 10:e0132221.

**Supplemental Table 3 Primers used in this study**

| **Prime name** | **Suquence** | **Description** |
| --- | --- | --- |
| 20291bF | aatttttagacttaagggcgCAAAGTTTACATATTTATTTCAGACAACG | The forward PCR primer for Amplification of *tcdB* promoter of R20291, *in vivo* study |
| 20291bR | tcttctcctttagataccatAAAATTTTCTCCTTTACTATAATATTTTTATTGA | The reverse PCR primer for Amplification of *tcdB* promoter of R20291, *in vivo* study |
| 20291RF | aatttttagacttaagggcgGGTTTCTAGATTTCATAAAAGATACTATTTTAGT | The forward PCR primer for Amplification of *tcdR* promoter of R20291, *in vivo* study |
| 20291RR | tcttctcctttagataccatAAAATCATCCTCTCTTATATTTATAATGATGA | The reverse PCR primer for Amplification of *tcdR* promoter of R20291, *in vivo* study |
| AN190bF | aatttttagacttaagggcgGAATGTTTACATATTTATTTCAGGCAAC | The forward PCR primer for Amplification of *tcdB* promoter of AN190, *in vivo* study |
| AN190bR | tcttctcctttagataccatAAAATTTTCTCCTTTACTATAATATTTTTATTAAA | The reverse PCR primer for Amplification of *tcdB* promoter of AN190, *in vivo* study |
| AN190RF | aatttttagacttaagggcgAAAAAGTTTTTTAGACTTCATAAAAGATACTATT | The forward PCR primer for Amplification of *tcdR* promoter of AN190, *in vivo* study |
| AN190RR | tcttctcctttagataccatAAAATCATCCCCTCTTATATTTATAATGA | The reverse PCR primer for Amplification of *tcdR* promoter of AN190, *in vivo* study |
| AN164bF | aatttttagacttaagggcgATTTTATATAGAACAAAGTTTACATATTTATTTCA | The forward PCR primer for Amplification of *tcdB* promoter of AN164, *in vivo* study |
| AN164bR | tcttctcctttagataccatAAAATTTTCTCCTTTACTATAATATTTTTACATC | The reverse PCR primer for Amplification of *tcdB* promoter of AN164, *in vivo* study |
| TcdR20291pUC-F53.2 | acctgacgtctaagaaaccaGGTTTCTAGATTTCATAAAAGATACT | The forward PCR primer for Amplification of *tcdB* promoter of R20291, *in vitro* study |
| TcdR20291pUC-R52.8 | ATATTTTTTGCATCTTCCATAAAATCATCCTCTCTTATATTTATAAT | The reverse PCR primer for Amplification of *tcdB* promoter of R20291, *in vitro* study |
| TcdR630pUC-F53.2 | acctgacgtctaagaaaccaGGTTTCTAGATTTCATAAAAGATACT | The forward PCR primer for Amplification of *tcdB* promoter of 630, *in vitro* study |
| TcdRAN190pUC-F55.8 | acctgacgtctaagaaaccaAAAAAGTTTTTTAGACTTCATAAAAGA | The forward PCR primer for Amplification of *tcdB* promoter of AN190, *in vitro* study |
| TcdRAN190pUC-R56.4 | TATTTTTTGCATCTTCCAT AAAATCATCCCCTCTTATATTTATAAT | The reverse PCR primer for Amplification of *tcdB* promoter of AN190, *in vitro* study |
| TcdRAN164pUC-F54.5 | acctgacgtctaagaaaccaAAAGGTATCTAGATTTCATAAAAAATAT | The forward PCR primer for Amplification of *tcdB* promoter of AN64, *in vitro* study |
| TcdRAN164pUC-R55.6 | ATATTTTTTGCATCTTCCATAAAATCATCCTCTCTTATTTTATAATG | The reverse PCR primer for Amplification of *tcdB* promoter of AN164, *in vitro* study |
| TcdB20291pUC-F60.4 | acctgacgtctaagaaaccaCAAAGTTTACATATTTATTTCAGACAACG | The forward PCR primer for Amplification of *tcdB* promoter of R20291, *in vitro* study |
| TcdB20291pUC-R59.8 | ATATTTTTTGCATCTTCCATAAAATTTTCTCCTTTACTATAATATTTTTATTG | The reverse PCR primer for Amplification of *tcdB* promoter of R20291, *in vitro* study |
| TcdB630pUC-F56.9 | Acctgacgtctaagaaacca ATTTTATATAGAACAAAGTTTACATATTTATTT | The forward PCR primer for Amplification of *tcdB* promoter of 630, *in vitro* study |
| TcdB630pUC-R57 | ATATTTTTTGCATCTTC CATAAAATTTTCTCCTTTACTATAATATTTTTAC | The reverse PCR primer for Amplification of *tcdB* promoter of 630, *in vitro* study |
| TcdBAN190pUC-F57.7 | ATATTTTTTGCATCTTC CATAAAATTTTCTCCTTTACTATAATATTTTTATT | The forward PCR primer for Amplification of *tcdB* promoter of AN190, *in vitro* study |
| TcdBAN190pUC-R58 | agggaaaccgttgtggtctcAAAATTTTCTCCTTTACTATAATATTTTTATTAAA | The reverse PCR primer for Amplification of *tcdB* promoter of AN190, *in vitro* study |
| TcdBAN164pUC-F58.2 | acctgacgtctaagaaacca ATTTTATATAGAACAAAGTTTACATATTTATTTC | The forward PCR primer for Amplification of *tcdB* promoter of AN164, *in vitro* study |
| TcdBAN164pUC-R57 | ATATTTTTTGCATCTTC CATAAAATTTTCTCCTTTACTATAATATTTTTAC | The reverse PCR primer for Amplification of *tcdB* promoter of AN164, *in vitro* study |
| Pthl-R | acgctacgatcaagcttAACTTAATTATACCCACTATTATTATTTTTATCAATATATTTTGTTAAAAATGGTTTCTTAGACGTCAGGTGG | The reverse PCR primer for amplification of *Clostridium* promoter P_thl_, *in vivo* study |
| p7918-F | attgacgcgtattgggatTTTTTAACAGACGATCTTGATAAAATTAAAAATAGTCCGTATAATTAAGGAGAGACCACAACGGTTTCCCTC | The forward PCR primer for amplification of *Clostridium* promoter P_%79-18_, *in vivo* study |
| p7918-R | ATCCCAATACGCGTCAATTCA | The reverse PCR primer for amplification of *Clostridium* promoter P_%79-18_, *in vivo* study |
| p7920-F | attgacgcgtattgggatTGTATAACAAAAAACATTGATAAAAATAAATCGTGACCGTATAATTAACTTGAGACCACAACGGTTTCCCTC | The forward PCR primer for amplification of *Clostridium* promoter P_%79-20_, *in vivo* study |
| p7926-R | acgctacgatcaagcttAACTTAATTATACACACTATTATAATTTTTATCAATATATTTTGTTAAAAGTGGTTTCTTAGACGTCAGGTGG | The reverse PCR primer for amplification of *Clostridium* promoter P_%79-26_, *in vivo* study |
| p7928-F | attgacgcgtattgggatATTTTAACAAAATATATTGATACAAATACTAATAGTGGGTATAATTATATCGAGACCACAACGGTTTCCCTC | The forward PCR primer for amplification of *Clostridium* promoter P_%79-28_, *in vivo* study |
| P7933-R | acgctacgatcaagcttAACTTAATTATACACAGCATGGTAAGTTTTATCAATATATTTTGTAATTAATGGTTTCTTAGACGTCAGGTGG | The reverse PCR primer for amplification of *Clostridium* promoter P_%79-33_, *in vivo* study |
| p7951-F | attgacgcgtattgggatTTTTTAACACCATAAATTGATAAAAATTATAATAGTGGGTATAATTAGGATGAGACCACAACGGTTTCCCTC | The forward PCR primer for amplification of *Clostridium* promoter P_%79-51_, *in vivo* study |
| CPthl-F | aatttttagacttaagggcgTTTTTAACAAAATATATTGATAAAAATAATAATAGTG | The forward PCR primer for amplification of *Clostridium* promoter P_thl_, *in vitro* study |
|  |  |  |
| CP18-F | aatttttagacttaagggcgTTTTTAACAGACGATCTTGATAAAATTAA | The forward PCR primer for amplification of *Clostridium* promoter P_%79-18_, *in vitro* study |
|  |  |  |
| CP20-F | aatttttagacttaagggcgCTGTATAACAAAAAACATTGATAAAAATAAAT | The forward PCR primer for amplification of *Clostridium* promoter P_%79-20_, *in vitro* study |
|  |  |  |
| CP26-F | aatttttagacttaagggcgCTTTTAACAAAATATATTGATAAAAATTATAATAGTG | The forward PCR primer for amplification of *Clostridium* promoter P_%79-26_, *in vitro* study |
|  |  |  |
| CP28-F | aatttttagacttaagggcgATTTTAACAAAATATATTGATACAAATACTAATAGTG | The forward PCR primer for amplification of *Clostridium* promoter P_%79-28_, *in vitro* study |
|  |  |  |
| CP33-F | aatttttagacttaagggcgTTAATTACAAAATATATTGATAAAACTTACCATG | The forward PCR primer for amplification of *Clostridium* promoter P_%79-33_, *in vitro* study |
| CP51-F | aatttttagacttaagggcgTTTTTAACACCATAAATTGATAAAAATTATAA | The forward PCR primer for amplification of *Clostridium* promoter P_%79-51_, *in vitro* study |
| CPromoter-R | tcttctcctttagataccatATGTATATCTCCTTCTTAAAGTTAAAC | The reverse PCR primer for amplification of all *Clostridium* promoters, *in vitro* study |
